# Long noncoding RNA NR2F1-AS1 stimulates the tumorigenic behavior of non-small cell lung cancer cells by sponging miR-363-3p to increase SOX4

**DOI:** 10.1515/med-2021-0403

**Published:** 2021-12-16

**Authors:** Luming Jin, Chaoyang Chen, Lipeng Huang, Qingyu Sun, Liang Bu

**Affiliations:** Department of Thoracic Surgery, Xiamen University Institute of Chest and Lung Disease, Xiang’an Hospital of Xiamen University, Xiamen, China

**Keywords:** non-small cell lung cancer, glycolysis, NR2F1-AS1, miR-363-3p, SOX4

## Abstract

Long noncoding RNA (lncRNA), specifically the upregulation of lncRNA NR2F1 antisense RNA 1 (NR2F1-AS1), has been involved in the progression of non-small cell lung cancer (NSCLC), but the mechanisms that underlie this remain unclear. In this study, the expression of NR2F1-AS1, miR-363-3p, and SOX4 was assessed in NSCLC cells. A loss-of-function assay was used to measure the tumorigenicity of NSCLC cells. The glycolysis and glutamine metabolism of NSCLC cells was also measured via extracellular acidification rate, consumption of glucose and glutamine, and production of lactate and ATP. The relationships among NR2F1-AS1, miR-363-3p, and SOX4 were detected via dual-luciferase reporter assay. HK-2, GLS1, and SOX4 levels were also analyzed. We found that both NSCLC tissues and cells had higher levels of NR2F1-AS1. Silencing of NR2F1-AS1 inhibited the tumorigenicity of cells *in vitro* and reduced the glycolysis and glutamine metabolism of NSCLC cells. Regarding its mechanism, NR2F1-AS1 positively regulated the SOX4 level by sponging miR-363-3p. Furthermore, miR-363-3p inhibition or SOX4 overexpression reversed the repressing role of sh-NR2F1-AS1 in the tumorigenicity of NSCLC cells. In summary, NR2F1-AS1 promotes the tumorigenicity of NSCLC cells by regulating miR-363-3p/SOX4.

## 1 Introduction

Lung cancer, one of the most serious cancers globally, is broadly classified into small cell lung cancer (SCLC) and non-small cell lung cancer (NSCLC). Clinically, most lung cancer patients are diagnosed as NSCLC [1,2]. Despite the current chemotherapeutic and radiotherapeutic treatments, many NSCLC patients still have a poor prognosis due to the lack of a reliable treatment strategy [3]. More recently, the development of targeted therapy and immunotherapy has prolonged the survival of NSCLC patients [4], but treatment outcomes remain unsatisfactory. Therefore, it is extremely vital to find novel therapeutic strategies for NSCLC patients. Emerging evidence has demonstrated that the activation of NSCLC cells is related to glucose consumption and metabolism [5,6]. Inhibiting glucose metabolism can also delay NSCLC progression, which can potentially be a new reliable strategy for NSCLC therapy [7]. Although more knowledge has emerged regarding the metabolic reprogramming of NSCLC, the mechanisms underlying NSCLC glycolysis remain obscure.

Long noncoding RNAs (lncRNAs) include many transcripts that lack protein-coding ability [8,9]. Until now, lncRNAs have now been closely implicated in several biological roles, such as transcriptional regulation [10], RNA processing [11], epigenetic modification [12], and posttranslational modification [13]. Aberrant lncRNA expression has vital functions during tumorigenesis and glycolysis [14,15]. In fact, various dysregulations of lncRNA have been found regarding the growth, metastasis, and development of NSCLC [16,17]. For example, the elevated expression of hox transcript antisense intergenic RNA in NSCLC tissues is associated with a shorter overall survival time [18,19]. By controlling TDG-mediated acetylation, LINC00467 increased cell growth and metastasis through the Akt pathway in NSCLC cells [20]. He et al. found that lncRNA NNT-AS1 acts as a sponge in regulating YAP1 levels through competition for miR-22-3p, thereby promoting the progression of NSCLC [21]. LINC00243 elevates NSCLC cell proliferation and glycolysis via overexpression of PDK4 by sponging miR-507 [22]. Therefore, tumor-specific lncRNAs are the potential targets for the development of novel strategies against NSCLC [23,24].

A few reports have linked lncRNA NR2F1 antisense RNA 1 (NR2F1-AS1) to tumorigenesis and tumor progression [25,26,27]. However, the extent of the pro-oncogenic effect of NR2F1-AS1 on NSCLC is limited [28]. In this study, we found that NR2F1-AS1 induces tumorigenicity and glycolysis in NSCLC cells. We also demonstrated that NR2F1-AS1 knockdown or miR-363-3p mimics can repress glycolysis in NSCLC cells. Moreover, we observed that NR2F1-AS1 exerts this pro-oncogenic role via miR-363-3p/SOX4. Our data may provide new evidence for targeting NR2F1-AS1 as a promising therapeutic approach for NSCLC.

## 2 Materials and methods

### 2.1 Clinical specimen collection

A total of 20 paired NSCLC tumors and normal samples were obtained from Xiang’an Hospital. All subjects gave written informed consent. This study was approved by the Ethics Committee of the Xiang’an Hospital. All samples were stored in liquid nitrogen for use.

### 2.2 Cell lines and transfection

Human normal bronchial epithelial cell line 16HBE, A549, and H522 cells were purchased from Shanghai Cellular Research Institute and cultured in RPMI-1640 medium (ScienCell, Beijing, China) with 10% fetal bovine serum (FBS) in a 37°C atmosphere with 5% CO_2_.

The shRNA of NR2F1-AS1 (sh-NR2F1-AS1#1-3), miR-363-3p mimic and inhibitor, pcDNA SOX4 overexpression plasmid, and the NC controls were obtained from Genepharma (Shanghai, China). The cells were transfected with lipofectamine 3000 reagent for 48 h.

### 2.3 Quantitative real-time PCR (qRT-PCR)

The trizol reagent was utilized, and cDNA was synthesized using the cDNA synthesis kit (Roche, Basel, Switzerland). One Step SYBR PrimeScript RT-PCR kit was used for qRT-PCR. The primers were listed as follows: NR2F1-AS1 (sense: 5′-CATGCCGTGATGTAAGCTGC-3′; antisense: 5′-GCGACTGTTTCACCTCTCCA-3′), miR-363-3p (sense: 5′-GCCGAGAATTGCACGGTATC-3′; antisense: 5′-CTCAACTGGTGTCGTGGA-3′), U6 (sense: 5′-CTCGCTTCGGCAGCACA-3′; antisense: 5′-AACGCTTCACGAATTTGCGT-3′), and β-actin (sense: 5′-CTCGCCTTTGCCGATCC-3′; antisense: 5′-TCTCCATGTCGTCCCAGTTG-3′).

### 2.4 Cell counting kit 8 (CCK8) and clonogenic assay

NSCLC cells were planted into a 96-well culture. At the indicated time, the cells were treated with CCK8 (Beyotime, Shanghai, China). The OD value was determined at 450 nm using a microplate reader.

For clonogenic assay, NSCLC cells were planted in 2.5 cm dishes for 2 weeks at 37°C. The colonies were dyed with crystal violet (Beyotime, Shanghai, China) and counted via a microscope.

### 2.5 Transwell assay

The upper transwell unit precoated with matrigel (Corning, China) was used in the invasion assay and not in the migration assay. Cells were planted in the top unit coated with Dulbecco’s modified Eagle’s medium. The RPMI-1640 medium and 15% FBS were supplied into the lower unit. After 12 h, the cells in the lower surface were stained with DAPI and counted.

### 2.6 Measurement of glycolysis and extracellular acidification rate (ECAR)

Glucose consumption and lactate accumulation were measured using the Glucose Assay Kit and Lactate Assay Kit (Abcam, CA, USA) according to the manufacturer’s instructions.

Cells were consecutively treated with glucose, oligomycin, and 2-deoxyglucose. The ECAR of cells was detected using the Glycolysis Rate Determination Kit and evaluated using the Seahorse XFe Extracellular Flux Analyzer (Seahorse Bioscience Inc., Billerica, MA, USA).

### 2.7 Western blot analysis

Proteins were separated from the samples using the radioimmunoprecipitation assay buffer lysis buffer. The samples were resolved using 10% sodium dodecyl sulfate polyacrylamide gel electrophoresis gel and transferred to nitrocellulose membranes. The blots were incubated with the primary antibodies HK-2, 1, GLS1, and SOX4, followed by the secondary antibody. The blots were identified using enhanced chemiluminescence system.

### 2.8 Dual-luciferase reporter assay

The sequences of NR2F1-AS1 and SOX4 3'UTR were cloned into wild-type (WT) or mutate-type vectors and cotransfected with either miR-363-3p mimic or miR-NC into cells. The Dual-Luciferase Reporter Assay System was used to calculate the luciferase activity.

### 2.9 Statistical analysis

Measurements are presented as the mean ± standard deviation. The *t*-test was conducted for two groups, whereas one-way analysis of variance followed by a *post hoc* test were conducted for three or more groups using the SPSS 19.0 software (IBM, Armonk, NY, USA). Statistical significance was set at *P* < 0.05.

## 3 Results

### 3.1 Expression of NR2F1-AS1 was upregulated in NSCLC tissues and cell lines

**Figure 1:**
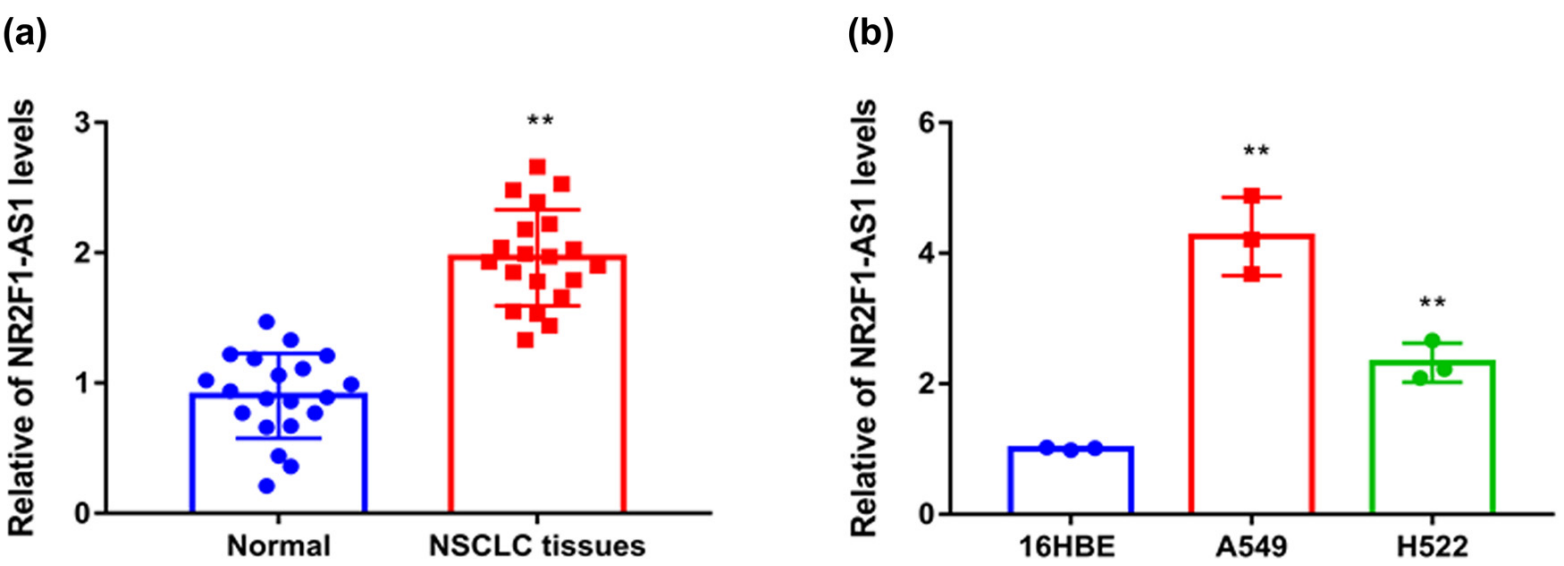
The expression of NR2F1-AS1 in NSCLC tissues and cell lines: (a) the expression of NR2F1-AS1 in the paired NSCLC tumor samples and adjacent samples were measured via qRT-PCR and (b) the NR2F1-AS1 expression in 16HBE cells and NSCLC cell lines (A549 and H522) were measured via qRT-PCR. **P < 0.01.

To observe the expression of NR2F1-AS1 in NSCLC tissues and cell lines, the qRT-PCR assay was performed. Expression of NR2F1-AS1 was notably higher in NSCLC samples than that in normal tissues (Figure 1a). Particularly, NR2F1-AS1 levels were enhanced in A549 and H522 cells than that in 16HBE cells (Figure 1b). So, NR2F1-AS1 expression was upregulated in NSCLC tissues and cell lines.

### 3.2 NR2F1-AS1 had an oncogenic effect on the progression of A549 and H522 cells

**Figure 2:**
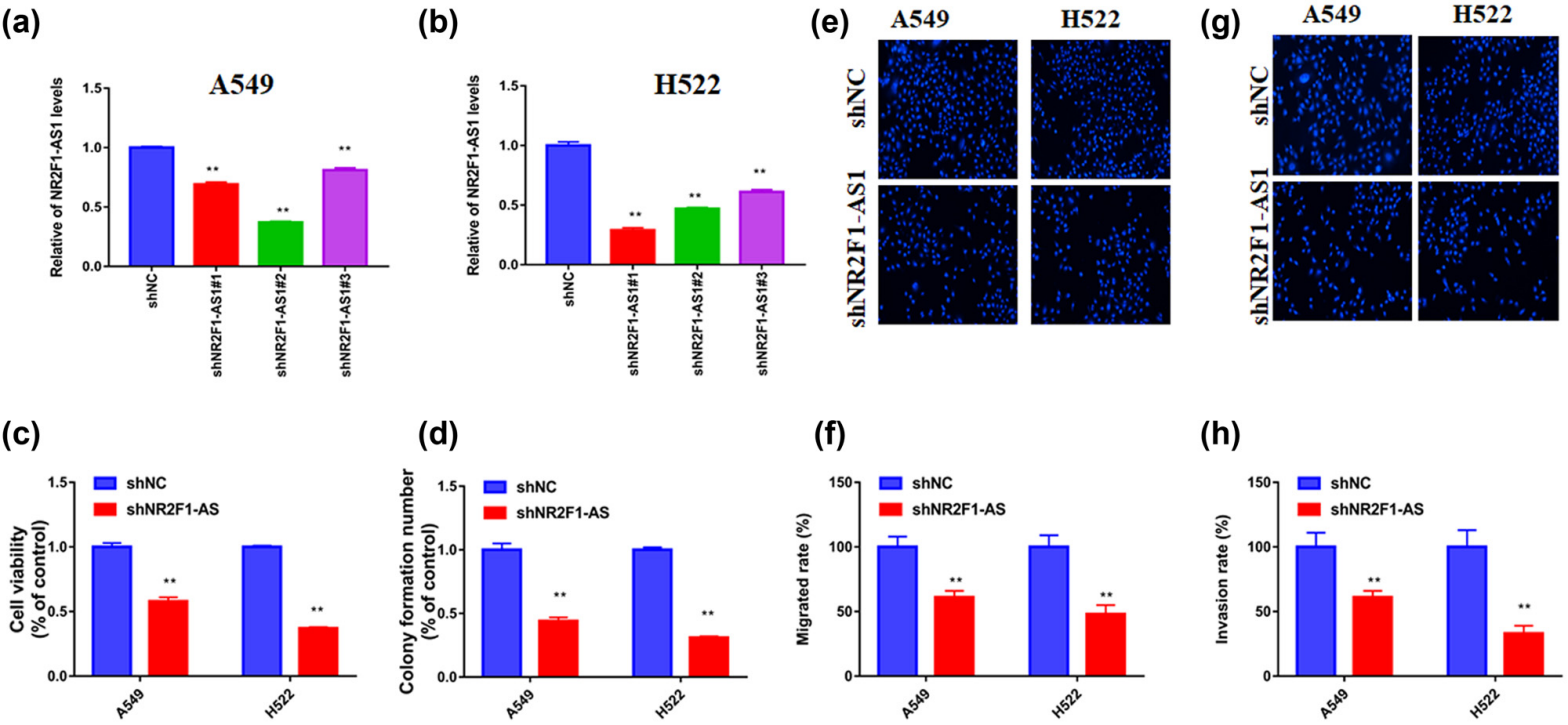
NR2F1-AS1 silencing blocked NSCLC progression *in vitro*. A549 and H522 cells were transfected with sh-NC or sh-NR2F1-AS1. (a and b) The level of NR2F1-AS1 was assessed to check the transfection efficiency of shRNA1-3. (c) The CCK8 assay, (d) colony formation assay, (e and f) transwell assay, and (g and h) invasion assay were operated to determine NSCLC progression *in vitro*. **P < 0.01.

To determine the biological role of NR2F1-AS1 in NSCLC cells, three shRNAs of NR2F1-AS1 were subjected to a loss-of-function test. Then, sh-NR2F1-AS1#2 were used. After treatment with sh-NR2F1-AS1, NR2F1-AS1 expression was effectively hindered in both A549 and H522 cells (Figure 2a and b). Both CCK8 assay and colony formation data showed that the downregulation of NR2F1-AS1 could reduce growth in NSCLC cells (Figure 2c and d). Moreover, transwell and invasion assays observed that the silencing of NR2F1-AS1 had an antimigrative effect on NSCLC cells (Figure 2e–h). Altogether, these findings confirm that NR2F1-AS1 has an oncogenic effect on the progression of NSCLC cells.

### 3.3 Downregulation of NR2F1-AS1 prevented glycolysis in NSCLC cells

**Figure 3:**
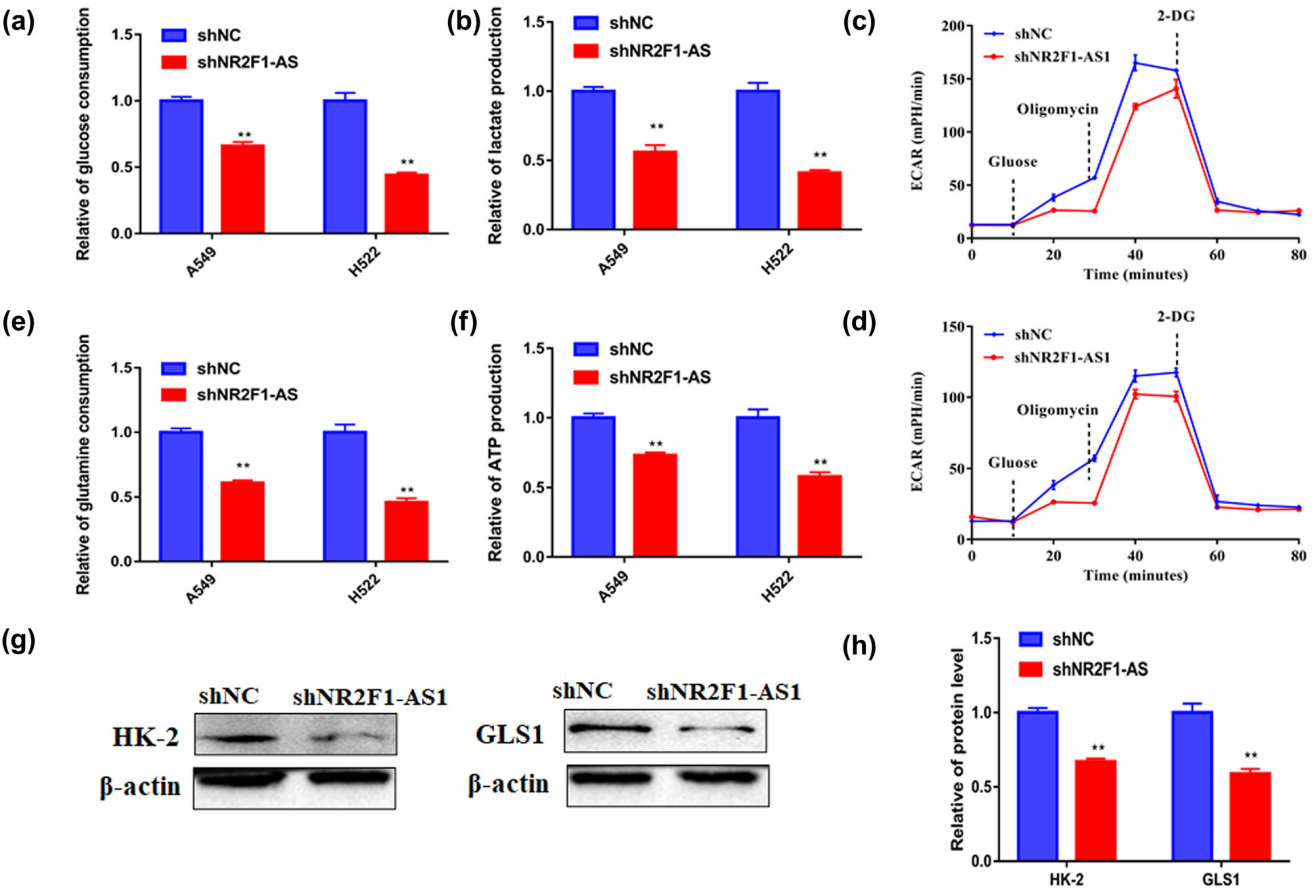
NR2F1-AS1 silencing limited the NSCLC cells’ glycolysis and glutamine metabolism. A549 and H522 cells were transfected with sh-NC or sh-NR2F1-AS1. (a–d) The glucose consumption, lactate production, and ECAR were quantified to assess cell glycolysis. (e and f) Glutamine consumption and ATP release were determined by the corresponding assay. (g and h) Western blot analysis was presented to assess HK-2 and GLS1 expression. **P < 0.01.

Inhibiting glycolysis is a new therapeutic strategy for NSCLC. As shown in Figure 3a and b, NR2F1-AS1 knockdown notably diminished the glucose uptake and lactate production of A549 and H522 cells, demonstrating that NR2F1-AS1 is positively correlated with the glycolysis of NSCLC cells. Similarly, reduction of NR2F1-AS1 inhibited glycolysis in NSCLC cells as determined using ECAR (Figure 3c and d). Moreover, the glutamine consumption and ATP levels of A549 and H522 cells were blocked after the reduction of NR2F1-AS1 (Figure 3e and f), implying that NR2F1-AS1 is positively correlated with the glutamine metabolism of NSCLC cells. Furthermore, the expression of glycolysis rate-limiting enzyme HK-2 and glutamine hydrolase GLS1 were clearly diminished by sh-NR2F1-AS1 (Figure 3g and h). Thus, NR2F1-AS1 inhibition prevented glycolysis in NSCLC cells.

### 3.4 NR2F1-AS1 could sponge miR-363-3p

**Figure 4:**
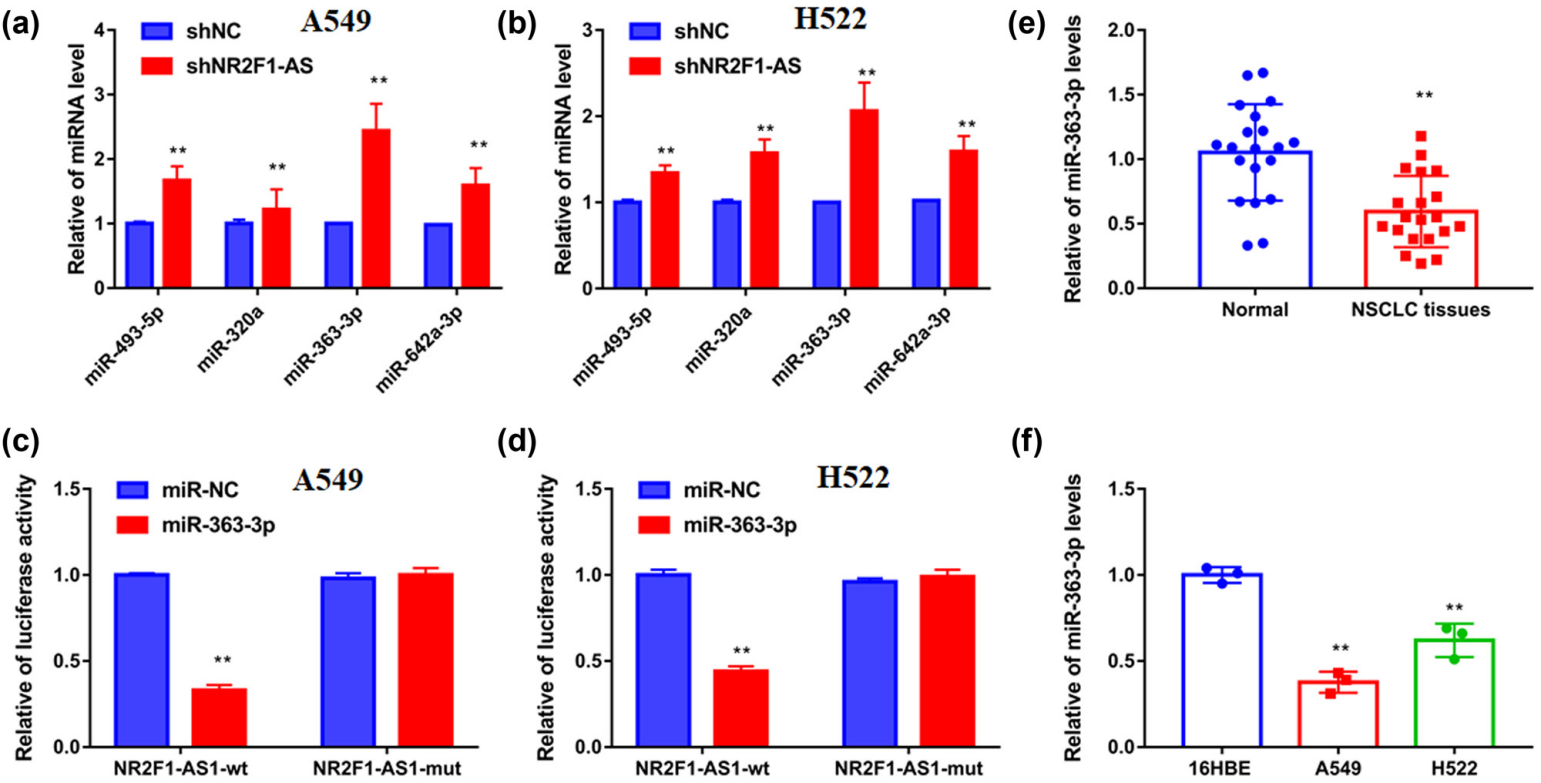
NR2F1-AS1 served as miR-363-3p sponge in NSCLC cells. (a and b) By transfecting with sh-NC or sh-NR2F1-AS1, the expression of miR-493-5p, miR-320a, miR-363-3p, and miR-642a-3p was assessed in A549 and H522 cells by qRT-PCR. (c and d) The connections between miR-363-3p and NR2F1-AS1 were presented by dual-luciferase reporter assay. (e) The level of miR-363-3p in the paired NSCLC samples and adjacent samples was tested by qRT-PCR. (f) The expression of miR-363-3p in BEAS-2B cells and NSCLC cell lines (A549 and H522) was tested by qRT-PCR. **P < 0.01.

The mechanism of NR2F1-AS1 was determined via software analysis, with four miRNAs – miR-493-5p, miR-320a, miR-363-3p, and miR-642a-3p – predicted as candidate miRNAs. After silencing of NR2F1-AS1, miRNA expression was tested, and we observed that the expression of miR-363-3p was the highest (Figure 4a and b). Thus, miR-363-3p was chosen for this study. Next, the dual-luciferase reporter data revealed that miR-363-3p mimics remarkably repressed luciferase activity of NR2F1-AS1 WT vector, but it did not affect the NR2F1-AS1 mutant vector (Figure 4c and d). Finally, a lower level of miR-363-3p was found in NSCLC samples (Figure 4e and f), which was the inverse of the expression pattern of NR2F1-AS1. Therefore, NR2F1-AS1 could sponge miR-363-3p expression.

### 3.5 NR2F1-AS1 promoted NSCLC progression by working as miR-363-3p sponging

**Figure 5:**
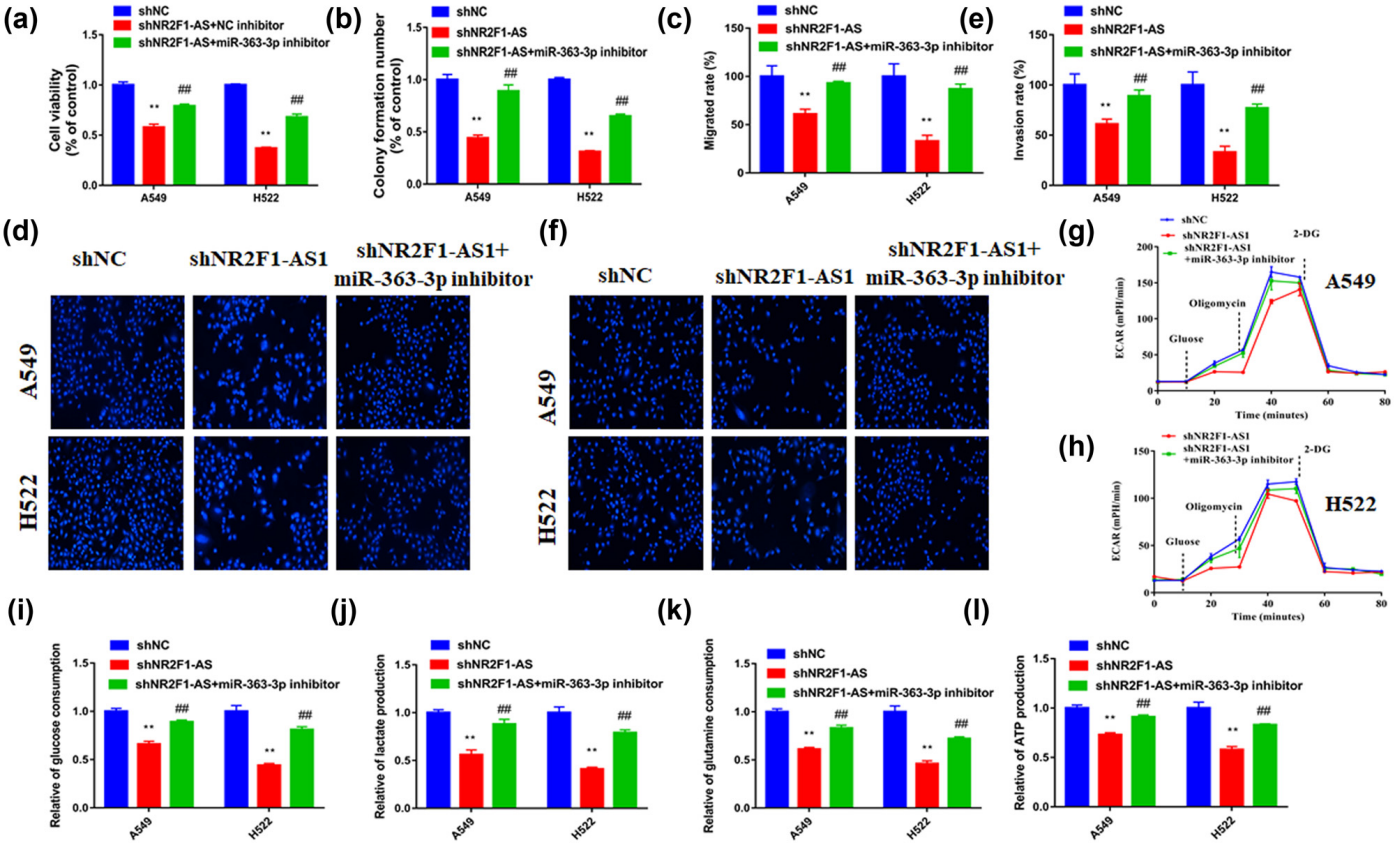
NR2F1-AS1 promoted NSCLC progression via sponging miR-363-3p. A549 and H522 cells were transfected with sh-NC, sh-NR2F1-AS1, sh-NR2F1-AS1 + anti-miR-NC, or sh-NR2F1-AS1 + anti-miR-363-3p. The (a) viability, (b) colony number, (c and d) migration, and (e and f) invasion of cells were determined using CCK8 assay, colony formation assay, and transwell assay. (g and h) Cell glycolysis, (i) glucose consumption, (j) lactate production, (k) glutamine consumption, and (l) ATP production were evaluated by the corresponding assay, respectively. **P < 0.01 compared with the sh-NC group. ^##^ *P* < 0.01 compared with sh-NR2F1-AS1 group.

To verify whether NR2F1-AS1 regulates NSCLC progression by modulating miR-363-3p expression, the sh-NR2F1-AS1 and miR-363-3p inhibitors were cotransfected into cells. The cell function analysis demonstrated that miR-363-3p inhibitor attenuated the inhibitory role of NR2F1-AS1 downregulation in the progression of A549 and H522 cells (Figure 5a–f). Furthermore, the repressing effect of sh-NR2F1-AS1 on ECAR, glucose consumption, lactate generation, glutamine consumption, and ATP production of NSCLC cells was abrogated via miR-363-3p inhibitor (Figure 5g–l). So, NR2F1-AS1 promoted NSCLC progression via miR-363-3p sponging.

### 3.6 Upregulation of SOX4 reversed the repressing effect of sh-NR2F1-AS1 on NSCLC progression

**Figure 6:**
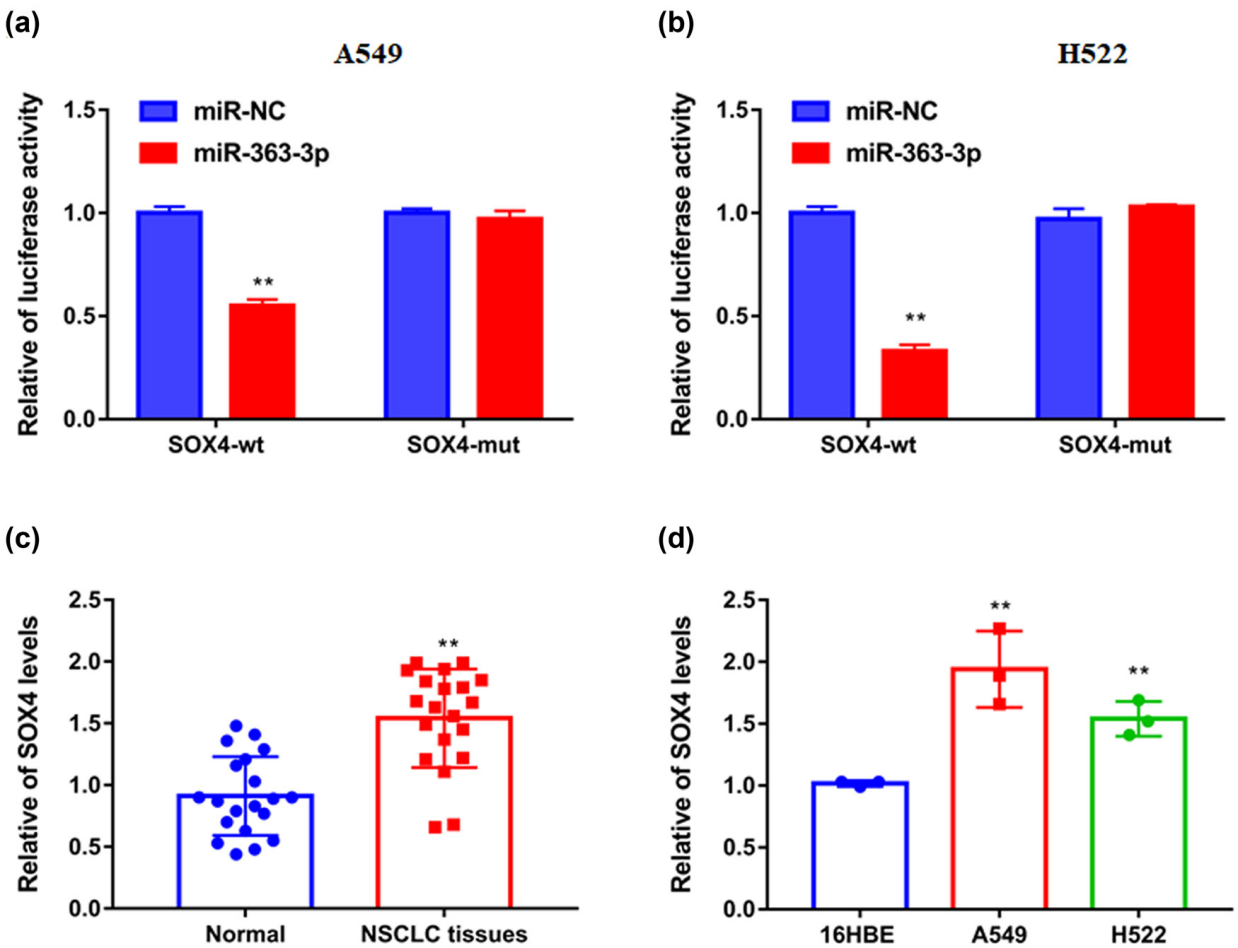
SOX4 was targeted by miR-363-3p. (a and b) The connections between SOX4 3'UTR and miR-363-3p were exhibited using dual-luciferase reporter assay. (c and d) The mRNA levels of SOX4 in the paired NSCLC samples and adjacent tissues, together with BEAS-2B cells and NSCLC cell lines (A549 and H522), were gaged by qRT-PCR. **P < 0.01.

Further tests revealed that miR-363-3p mimics reduce the luciferase activity of SOX4 3′UTR WT vector (Figure 6a and b). Likewise, a remarkably higher SOX4 mRNA level was found in NSCLC samples (Figure 6c and d).

**Figure 7:**
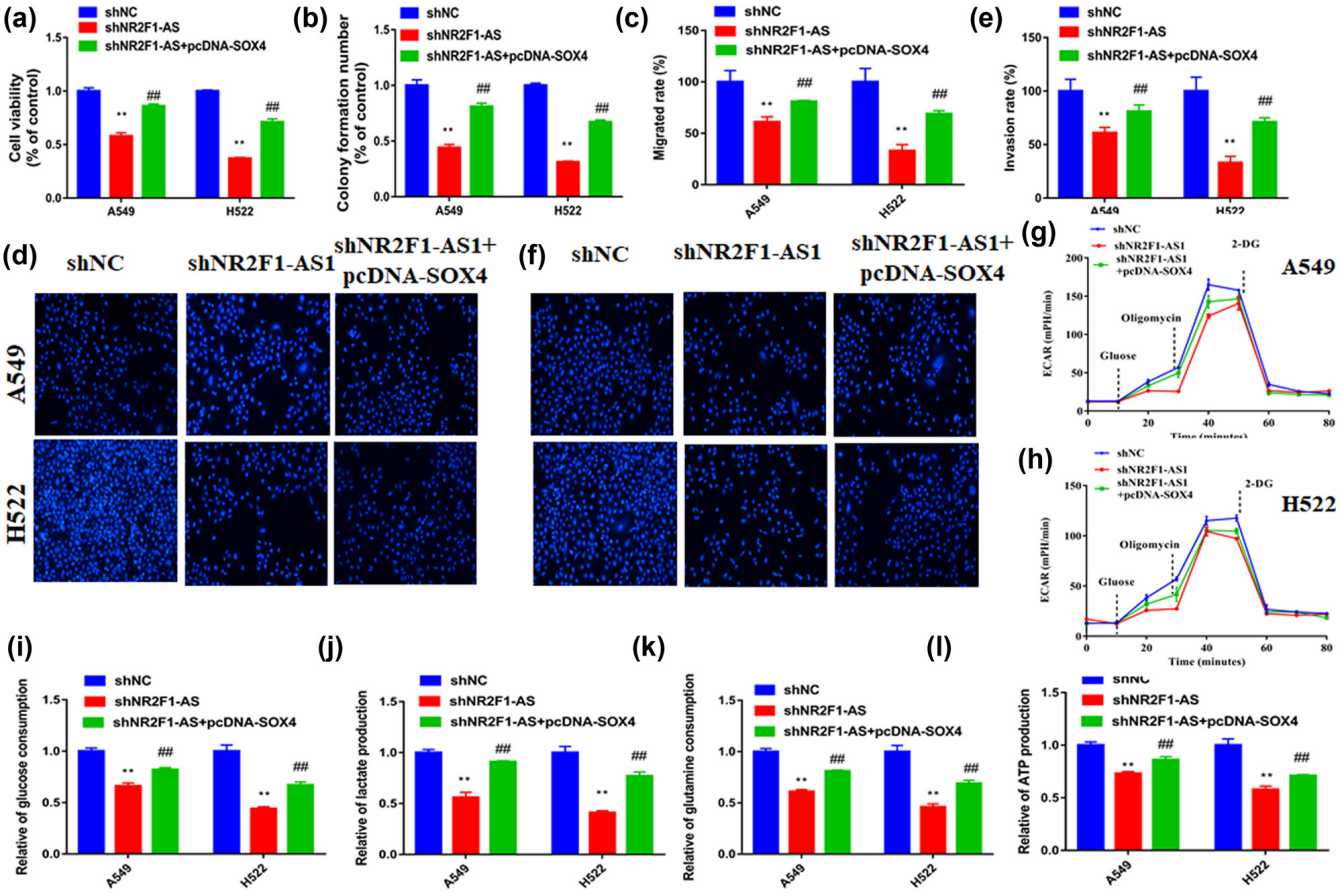
NR2F1-AS1 promoted NSCLC progression by SOX4 overexpression. A549 and H522 cells were transfected with sh-NC, sh-NR2F1-AS1, and sh-NR2F1-AS1 + pcDNA-SOX4. (a) The viability, (b) colony number, (c and d) migration, and (e and f) invasion of cells were determined using CCK8 assay, colony formation assay, and transwell assay. (g and h) Cell glycolysis, (i) glucose consumption, (j) lactate production, (k) glutamine consumption, and (l) ATP production were evaluated by the corresponding assay, respectively. **P < 0.01 compared with the sh-NC group. ^##^ *P* < 0.01 compared with sh-NR2F1-AS1 group.

Afterward, both sh-NR2F1-AS1 and pcDNA-SOX4 plasmid were cotransfected into cells. The viability, colony number migration, and invasion analysis demonstrated that pcDNA-SOX4 attenuates the repression effect of NR2F1-AS1 downregulation in the progression of A549 and H522 cells (Figure 7a–f). Furthermore, the repressing effect of NR2F1-AS1 downregulation on ECAR, glucose consumption, lactate generation, glutamine consumption, and ATP production of NSCLC cells was abrogated by pcDNA-SOX4 (Figure 7g–l). These findings suggest that NR2F1-AS1 modulates the tumorigenic behavior of NSCLC cells via the miR-363-3p/SOX4 axis.

## 4 Discussion

Reprogramming of the glucose metabolism has a vital role in tumorigenesis. During the development of tumor, glycolysis is strongly promoted in tumor cells, thereby augmenting cell aggressiveness and metastatic ability. Targeting inhibition of glycolysis is regarded as an effective strategy for the treatment of NSCLC. In this study, we determined the expression profile of NR2F1-AS1 in NSCLC patients and cell line. Furthermore, we found that NR2F1-AS1 regulates NSCLC cell oncogenicity including glycolysis by miR-363-3p/SOX4 axis.

There is increasing evidence that NR2F1-AS1 operates as an oncogene involved in the progression of NSCLC. Moreover, the expression of NR2F1-AS1 is a potential prognostic biomarker in the development of NSCLC. In accordance with this, we found that NR2F1-AS1 was overexpressed in NSCLC samples, and that the silencing of NR2F1-AS1 inhibits the glycolysis in NSCLC cells. Some studies have revealed that the overexpression of NR2F1-AS1 increased glycolysis in hepatocellular carcinoma cells [29]. Similarly, we found that NR2F1-AS1 is connected to the metabolic changes in NSCLC cells. An important finding is that the silencing of NR2F1-AS1 blocked glycolysis and cell progression of NSCLC cells.

Further, we predicted the four targeted miRNAs of NR2F1-AS1. We found that miR-363-3p is inversely related with the expression pattern of NR2F1-AS1. Other studies found that miR-363-3p plays a key role in other cancers, including hepatocellular carcinoma [30,31,32]. Moreover, we found that levels of miR-363-3p were lower in NSCLC cells, and that this was regulated by NR2F1-AS1. Because of the association between glycolysis and levels of miR-363-3p [33], we checked how miR-363-3p regulated glycolysis in NSCLC cells. Unsurprisingly, the miR-363-3p mimic partially blocked glycolysis in NSCLC cells. This implies that miR-363-3p mediates glycolysis in NSCLC cells. We also found that the miR-363-3p inhibitor overturned the repressing role of NR2F1-AS1 downregulation in glycolysis in NSCLC samples. In summary, deficiency of NR2F1-AS1 blocks glycolysis and tumorigenicity in NSCLC cells via miR-363-3p.

SOX4 is known to have oncogenic functions in several tumors, including glioblastoma and hepatocellular carcinoma. Similarly, higher level of SOX4 stimulated cell progression in NSCLC. Our report found that SOX4 was directly regulated by miR-363-3p in NSCLC cells, and that SOX4 was overexpressed in NSCLC samples.

It is well known that lncRNAs modulate the target mRNA expression by binding to miRNA. In this study, we determined whether NR2F1-AS1 affected the miR-615-3p/SOX4 signal in NSCLC cells. Interestingly, SOX4 level was positively associated with level of NR2F1-AS1, whereas it was negatively associated with level of miR-363-3p in NSCLC samples. Moreover, we found that NR2F1-AS1 induces cell progression in NSCLC samples, but these roles were overturned by miR-615-3p. Our findings imply that NR2F1-AS1 may regulate level of SOX4 via sponging miR-363-3p in NSCLC cells. Therefore, the NR2F1-AS1/miR-615-3p/SOX4 signal may be involved in the glycolysis in NSCLC cells.

In summary, this study showed that inhibition of NR2F1-AS1 suppressed glycolysis in NSCLC cells, and the regulation of miR-363-3p/SOX4 plays a role in this. These insights on NR2F1-AS1 could support its use as a potent treatment choice for NSCLC.

## References

[1] Arbour KC, Riely GJ. Systemic therapy for locally advanced and metastatic non-small cell lung cancer: a review. JAMA. 2019;322(8):764–74. 10.1001/jama.2019.11058 31454018

[2] Sung H, Ferlay J, Siegel RL, Laversanne M, Soerjomataram I, Jemal A, et al. Global cancer statistics 2020: GLOBOCAN estimates of incidence and mortality worldwide for 36 cancers in 185 countries. CA Cancer J Clin. 2021;71(3):209–49. 10.3322/caac.21660 33538338

[3] Kim YJ, Oremus M, Chen HH, McFarlane T, Shah D, Horton S. Real-world effectiveness of nivolumab in patients with non-small-cell lung cancer: a systematic review and meta-analysis. Future Oncol. 2020;16(27):2045–58. 10.2217/fon-2020-0248 32598192

[4] Shi Y, Chen W, Li C, Zhang Y, Bo M, Qi S, et al. Efficacy and safety of first-line treatments with immune checkpoint inhibitors plus chemotherapy for non-squamous non-small cell lung cancer: a meta-analysis and indirect comparison. Ann Palliat Med. 2021;10(3):2766–75. 10.21037/apm-20-1498 33549014

[5] Luengo A, Abbott KL, Davidson SM, Hosios AM, Faubert B, Chan SH, et al. Reactive metabolite production is a targetable liability of glycolytic metabolism in lung cancer. Nat Commun. 2019;10(1):5604. 10.1038/s41467-019-13419-4 PMC6898239 31811141

[6] Lee JS, Lee H, Lee S, Kang JH, Lee SH, Kim SG, et al. Loss of SLC25A11 causes suppression of NSCLC and melanoma tumor formation. EBioMedicine. 2019;40:184–97. 10.1016/j.ebiom.2019.01.036 PMC6413681 30686754

[7] Wang K, Huang W, Chen R, Lin P, Zhang T, Ni YF, et al. Di-methylation of CD147-K234 promotes the progression of NSCLC by enhancing lactate export. Cell Metab. 2021;33(1):160–73e6. 10.1016/j.cmet.2020.12.010 33406400

[8] Zhou H, Feng B, Abudoureyimu M, Lai Y, Lin X, Tian C, et al. The functional role of long non-coding RNAs and their underlying mechanisms in drug resistance of non-small cell lung cancer. Life Sci. 2020;261:118362. 10.1016/j.lfs.2020.118362 32871184

[9] Wilusz JE, Sunwoo H, Spector DL. Long noncoding RNAs: functional surprises from the RNA world. Genes Dev. 2009;23(13):1494–504. 10.1101/gad.1800909 PMC3152381 19571179

[10] Zheng F, Chen J, Zhang X, Wang Z, Chen J, Lin X, et al. The HIF-1alpha antisense long non-coding RNA drives a positive feedback loop of HIF-1alpha mediated transactivation and glycolysis. Nat Commun. 2021;12(1):1341. 10.1038/s41467-021-21535-3 PMC7910558 33637716

[11] Shi L, Magee P, Fassan M, Sahoo S, Leong HS, Lee D, et al. A KRAS-responsive long non-coding RNA controls microRNA processing. Nat Commun. 2021;12(1):2038. 10.1038/s41467-021-22337-3 PMC8016872 33795683

[12] Cao T, Jiang Y, Wang Z, Zhang N, Al-Hendy A, Mamillapalli R, et al. H19 lncRNA identified as a master regulator of genes that drive uterine leiomyomas. Oncogene. 2019;38(27):5356–66. 10.1038/s41388-019-0808-4 PMC6755985 31089260

[13] Ye T, Yang X, Liu H, Lv P, Ye Z. Long non-coding RNA BLACAT1 in human cancers. Onco Targets Ther. 2020;13:8263–72. 10.2147/OTT.S261461 PMC7445530 32903916

[14] Zeng SHG, Xie JH, Zeng QY, Dai SHH, Wang Y, Wan XM, et al. lncRNA PVT1 promotes metastasis of non-small cell lung cancer through EZH2-mediated activation of Hippo/NOTCH1 signaling pathways. Cell J. 2021;23(1):21–31. 10.22074/cellj.2021.7010 PMC7944120 33650817

[15] Chen D, Li Y, Wang Y, Xu J. LncRNA HOTAIRM1 knockdown inhibits cell glycolysis metabolism and tumor progression by miR-498/ABCE1 axis in non-small cell lung cancer. Genes Genomics. 2021;43(2):183–94. 10.1007/s13258-021-01052-9 33537917

[16] Li Y, Shen R, Wang A, Zhao J, Zhou J, Zhang W, et al. Construction of a prognostic immune-related LncRNA risk model for lung adenocarcinoma. Front Cell Dev Biol. 2021;9:648806. 10.3389/fcell.2021.648806 PMC8044985 33869203

[17] Jiang W, Kai J, Li D, Wei Z, Wang Y, Wang W. lncRNA HOXB-AS3 exacerbates proliferation, migration, and invasion of lung cancer via activating the PI3K-AKT pathway. J Cell Physiol. 2020;235(10):7194–203. 10.1002/jcp.29618 32039488

[18] Yuan S, Xiang Y, Guo X, Zhang Y, Li C, Xie W, et al. Circulating long noncoding RNAs act as diagnostic biomarkers in non-small cell lung cancer. Front Oncol. 2020;10:537120. 10.3389/fonc.2020.537120 PMC7793881 33425713

[19] Ren MM, Xu S, Wei YB, Yang JJ, Yang YN, Sun SS, et al. Roles of HOTAIR in lung cancer susceptibility and prognosis. Mol Genet Genomic Med. 2020;8(7):e1299. 10.1002/mgg3.1299 PMC7336741 32394637

[20] Zhu Y, Li J, Bo H, He D, Xiao M, Xiang L, et al. LINC00467 is up-regulated by TDG-mediated acetylation in non-small cell lung cancer and promotes tumor progression. Oncogene. 2020;39(38):6071–84. 10.1038/s41388-020-01421-w 32796958

[21] He W, Zhang Y, Xia S. LncRNA NNT-AS1 promotes non-small cell lung cancer progression through regulating miR-22-3p/YAP1 axis. Thorac Cancer. 2020;11(3):549–60. 10.1111/1759-7714.13280 PMC7049499 31923353

[22] Feng X, Yang S. Long non-coding RNA LINC00243 promotes proliferation and glycolysis in non-small cell lung cancer cells by positively regulating PDK4 through sponging miR-507. Mol Cell Biochem. 2020;463(1–2):127–36. 10.1007/s11010-019-03635-3 31595421

[23] Li S, Lin L. Long noncoding RNA MCF2L-AS1 promotes the cancer stem cell-like traits in non-small cell lung cancer cells through regulating miR-873-5p level. Env Toxicol. 2021;36(7):1457–65. 10.1002/tox.23142 33783940

[24] Wang X, Yu X, Wei W, Liu Y. Long noncoding RNA MACC1-AS1 promotes the stemness of nonsmall cell lung cancer cells through promoting UPF1-mediated destabilization of LATS1/2. Env Toxicol. 2020;35(9):998–1006. 10.1002/tox.22936 32401390

[25] Bai M, Wu ZZ, Huang YL, Ke J, Xu Q, Wang X. STAT3 activates the transcription of lncRNA NR2F1-AS1 to promote the progression of melanoma via regulating miR-493-5p/GOLM1 axis. J Gene Med. 2021;23(7):e3338. 10.1002/jgm.3338 33822440

[26] Zhang Q, Li T, Wang Z, Kuang X, Shao N, Lin Y. lncRNA NR2F1-AS1 promotes breast cancer angiogenesis through activating IGF-1/IGF-1R/ERK pathway. J Cell Mol Med. 2020;24(14):8236–47. 10.1111/jcmm.15499 PMC7348140 32548873

[27] Guo F, Fu Q, Wang Y, Sui G. Long non-coding RNA NR2F1-AS1 promoted proliferation and migration yet suppressed apoptosis of thyroid cancer cells through regulating miRNA-338-3p/CCND1 axis. J Cell Mol Med. 2019;23(9):5907–19. 10.1111/jcmm.14386 PMC6714216 31304680

[28] Zhang C, Wu S, Song R, Liu C. Long noncoding RNA NR2F1-AS1 promotes the malignancy of non-small cell lung cancer via sponging microRNA-493-5p and thereby increasing ITGB1 expression. Aging (Albany NY). 2020;13(5):7660–75. 10.18632/aging.103564 PMC7993723 32784268

[29] Li X, Li Y, Bai S, Zhang J, Liu Z, Yang J. NR2F1-AS1/miR-140/HK2 axis regulates hypoxia-induced glycolysis and migration in hepatocellular carcinoma. Cancer Manag Res. 2021;13:427–37. 10.2147/CMAR.S266797 PMC7815091 33488124

[30] Geng Q, Li Z, Li X, Wu Y, Chen N. LncRNA NORAD, sponging miR-363-3p, promotes invasion and EMT by upregulating PEAK1 and activating the ERK signaling pathway in NSCLC cells. J Bioenerg Biomembr. 2021;53(3):321–32. 10.1007/s10863-021-09892-6 33742335

[31] Chang J, Gao F, Chu H, Lou L, Wang H, Chen Y. miR-363-3p inhibits migration, invasion, and epithelial-mesenchymal transition by targeting NEDD9 and SOX4 in non-small-cell lung cancer. J Cell Physiol. 2020;235(2):1808–20. 10.1002/jcp.29099 31332786

[32] Lou W, Ding B, Zhong G, Du C, Fan W, Fu P. Dysregulation of pseudogene/lncRNA-hsa-miR-363-3p-SPOCK2 pathway fuels stage progression of ovarian cancer. Aging (Albany NY). 2019;11(23):11416–39. 10.18632/aging.102538 PMC6932902 31794425

[33] Khuu C, Sehic A, Eide L, Osmundsen H. Anti-proliferative properties of miR-20b and miR-363 from the miR-106a-363 cluster on human carcinoma cells. Microrna. 2016;5(1):19–35. 10.2174/2211536605666160322151813 27001184

